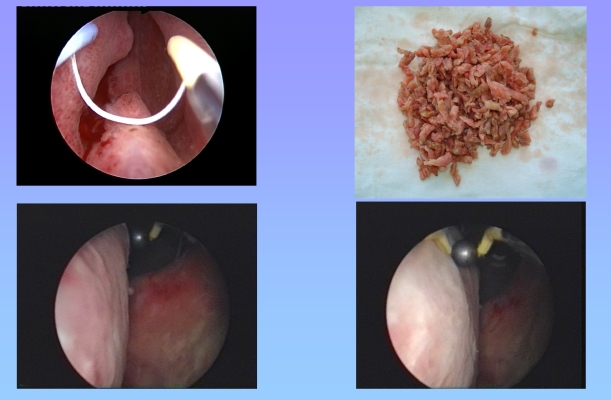# TURP for BPH. How Large is Too Large?

**Published:** 2010-11-25

**Authors:** C Persu, D Georgescu, I Arabagiu, V Cauni, C Moldoveanu, P Geavlete

**Affiliations:** Urology Department, ‘Saint John’ Clinical Emergency Hospital, Bucharest Romania

**Keywords:** BPH, TURP, open prostatectomy, EAU guidelines

## Abstract

BPH remains one of the most common disease that the urologist has to manage. The last decade brought numerous new techniques, aiming to improve the minimally invasive approach to BPH, but none had, for the moment, changed the place of TURP as the gold standard treatment for medium sized prostates.

Based on a large personal experience, the authors present a study in which TURP is used for prostates over 80ml, the cutoff point set by the guidelines of the European Association of Urology. The rationale for this study is that many situations require minimally invasive treatment, based on the express request of the patient, other conditions that makes open surgery very difficult or impossible, or the need for a quick discharge in an overcrowded service. The aim of the study was to prove that TURP is safe and effective even in larger prostates. The technique used is basically the classic one, with minor tactical alterations in some cases. Some cases required a two-stage approach, but offered good functional results after the first stage. The results proved that, with a good technique, a skilled urologist might achieve the same results by using TURP or open surgery for large sized prostates.

## Introduction

Benign prostatic hyperplasia is a progressive disease affecting an important number of men. The increase in public awareness and the numerous campaigns aiming to get men with LUTS to the specialist further increased the number of BPH cases, especially in those patients with mild and medium symptoms. The scientific and technological evolution led to the development of many non surgical treatment alternatives, limiting the indications of surgery. Despite all that, there still is a great number of patients with large prostates and severe symptoms or complications of BPH, requiring surgery.

TURP developed itself to become the gold standard of surgical treatment for medium sized prostates. The EAU guidelines, based on grade A evidence, recommends TURP for prostates between 35 and 80 ml. Over that limit, open surgery seems to remain the only option for treating BPH, according to available clinical evidence.

Still, in many cases, for the experienced urologist, TURP may represent a better alternative, first of all as a personal option, and second, as an option imposed by the need for short hospitalization and lower costs [1].

No need to remember the option of the patient, nowadays better informed and with many options ahead, who will ask for the least invasive treatment that will offer the best results, with minimal complications rate and that will allow him to restart his usual activities as soon as possible.

New technologies, including lasers, plasma vaporization, etc, are not suitable options for large prostates, and the lack of experience and clinical evidence may represent a drawback in recommending them to a vast number of patients [2].

In our country, the incidence of large sized prostates is higher than in other European countries, mainly due to the poor primary care offered in rural areas. This aspect is improving rapidly, but is still not uncommon to see patients with extremely severe LUTS, installed years ago, who never required or received any medical advice concerning their condition.

Based on the experience of our department, where more than 400 surgeries for BPH are performed every year, by several urologists, many of them successfully performing TURP even for prostates over 100ml, we performed a prospective study aiming to comparatively evaluate open surgery and TURP for prostates over 80ml.

## Patients and method

A single center, prospective, randomized study was proposed and performed, aiming to comparatively evaluate the results of TURP and open surgery, in terms of both intraoperative and postoperative features, for patients with prostatic volumes over 80 ml. This trial was presented to the local ethics committee and approved.

The patients that were included signed an informed consent properly describing the two techniques, and also the aims, method and expected benefit of the trial. Known hazards and the recommendations in the EAU Guidelines were also presented, along with the vast experience of our department in performing TURP for large prostates. The consent also stated that, in the TURP arm, the patient may have to undergo two interventions, in four weeks time, if local conditions will require.

The target population for the study was represented by all male subjects with prostates over 80ml and indication for surgery that could theoretically undergo both open and endoscopic intervention. The 200 subjects were randomized using sealed envelopes previously prepared and containing the name of the intervention – open surgery or TURP–in equal proportions. The study started in April 2008 and reached its target in November 2009.

The evaluation protocol included, in all cases, general clinical examination with DRE, blood tests, urine dipstick, abdominal ultrasonography, uroflowmetry, IPSS questionnaire and PSA measurement. In selected cases we performed IVP, KUB, cystoscopy and other tests relevant for the particular situation. IIEF–5 questionnaire was used to assess the sexual function of our patients. A three day bladder diary was recorded before the intervention. All the data from the evaluation was recorded in the patient's file.

The prostatic volume was calculated by ultrasonography, using the following formula: Volume = height X width X length X 0.52

Open surgery was performed using the suprapubic technique, with hypogastric incision and manual enucleation of the prostate. TURP was performed using the Storz Electroresection Unit, with the 24Fr working element and the classic loop. In some cases, several technical artifacts needed to be used, like axial movement of the resectoscope, suprapubic cystostomy during resection, starting the resection at 6 o'clock or 12 o'clock, etc. In some cases, according to the size of the prostatic lobes, the operative time and the intensity of the bleeding, the operator decided to stop the intervention after removing the right lobe and to continue the resection after four weeks. This attitude was considered normal, and not reported as a complication of the intervention. In these cases, data was analyzed globally for the two interventions, considering that, in the end, the patient was treated endoscopically, by one doctor.

The interventions were performed by six urologists, three for each group, with relevant expertise and personal options for performing either open or endoscopic surgery for BPH. The patients were randomly assigned to the doctors in the respective groups.

All the interventions were performed under spinal anesthesia. Patients were followed–up for three to six months, by evaluating the IPSS, Qmax, residual volume, urine infection and PSA. 

The study aimed to evaluate the total operative time, the amount of bleeding (evaluated by the need of transfusion), the mean time of catheterization, the total time of hospitalization, and the overall complication rate. The complications were rated minor and major, and assessed as intraoperative, postoperative (if they occurred during hospitalization) and complications during the follow up period.

The data was statistically analyzed using dedicated software, by means of Student's t test and chi square test, in order to evaluate the differences between values. The confidence level in this analysis reached 95%.

## Results

Data analysis showed that the two arms of the study contain population with similar demographic and urologic features, and that comparing the obtained data is relevant for our objective. Each group consisted of 100 patients.

In our series of TURP, the largest prostate treated measured 150 ml, with a mean value of 95 ml. In the open surgery group, the largest prostate was measured at 160 ml, with a mean value of 90 ml.

In 42 cases, in the TURP arm, the prostate was removed in two interventions, and the final result was consistent with the general features of the TURP arm. The main elements that lead to the decision of performing two interventions were: poor visibility due to bleeding or increased time of resection (over 45 minutes for one lobe). No particular complications or features were encountered in this subgroup.

The mean operative time was 50 minutes (40–75) for TURP and 65 minutes (45–85) for open surgery. In the subgroup of patients that underwent two TURPs, the time was analyzed for each intervention. We noticed that in all such cases, the second TUR took significantly less time than the first. Time was measured from the insertion of the resectoscope to the insertion of the catheter in the first arm, or from the incision to the last stitch for open surgery. 

For the TURP arm, we calculated a medium value of 1.8ml/min of resected tissue, a value above the average known from the literature.

The mean time of catheterization was 4 days after TURP and 9 days in the open prostatectomy arm. In 8 cases in the TURP arm, the patient developed acute urinary retention after the catheter was removed, so a new catheter was placed for 7 days. After that period, spontaneous micturition was obtained by 5 patients. 

Post TUR syndrome occurred in 2 patients, but, with adequate therapy measures, both patients recovered completely.

In the open prostatectomy arm, one patient died during the intervention due to massive myocardial infarction that did not respond to therapy. We consider this case as a complication of a known condition, and not of the particular urological technique.

Urinary incontinence was diagnosed after the removal of the catheter in 38 patients in the TURP arm and in 16 cases in the open surgery arm. In all cases, it was considered as a predictable outcome of the intervention and no therapeutic actions were taken. After three months, 5 patients in the TURP arm are still incontinent, compared to 3 patients in the open prostatectomy arm.

Overactive bladder syndrome was diagnosed during follow up in 7% of the cases treated by TURP and in 4.5% of the cases treated by open prostatectomy. In all cases, anticholinergic therapy was initiated. We noticed that the occurrence of such symptoms made all suffering patients unhappy with the result of their surgery.

De novo erectile dysfunction was diagnosed in 14.8% of the cases who underwent TURP and in 4% of the cases in the open surgery group. The statistical analysis could not establish a correlation between the prostatic volume and the occurrence of ED.

Urethral strictures, diagnosed due to the reappearance of obstructive symptoms, occurred in 3 patients in the TURP arm, and all needed surgical treatment. 

Osteitis pubis was diagnosed in 6 cases after open prostatectomy and is considered a disabling condition, although symptomatic relief is usually rapidly obtained by using analgesics and anti–inflammatory drugs.

Prostate cancer was diagnosed in 5 cases in the TURP arm and in 2 cases in the open prostatectomy arm. There is no particular explanation, in our opinion, for this difference, other than hazard.

## Discussions

In our clinical department are available many new modern treatment alternatives for BPH, including green laser, plasma vaporization, bipolar TUR, etc. The limited experience, higher costs, and the limited indications in patients with large prostates make the new techniques less useful for these patients, and not an option in the long run.

On the other hand, the vast experience with large prostates treated by open or endoscopic surgery allows us a more flexible approach in indicating TURP for a prostate that, according to the EAU Guidelines, is more appropriate for open surgery. In this decision, a great role is played by the patient, who, aware of the many new developments in medicine, asks for non–scaring surgery [3].

There is enough data in the literature to support the decision of performing TURP for a large prostate, in terms of safety and efficacy. Unfortunately, most studies compare TURP for small prostates with open prostatectomy for large prostates, so the results may be interpreted both as a difference between techniques or between series of patients [4].

The costs of the interventions may be different from one country to another, and that is why we did not try to compare this aspect with data from other studies.

An important issue is that contemporary series of open prostatectomies are rare, and so are the patients with prostates over 80 ml, especially in Western countries. Although there is not enough epidemiological data to support the affirmation, we may say that the incidence of such prostates is significantly higher in Eastern European countries, and the experience of large medical centers in these countries is quite relevant [5].

A multicentric, international study published in 1989 by Roos, comparing open prostatectomy and TURP concludes that endoscopy is less effective in terms of symptomatic relief and might associate a higher long–term mortality. Nevertheless, the authors did not perform a correlation of the prostatic volume with the findings of their study [6]. 

A retrospective study on over 5000 patients treated for BPH by open surgery concludes that the technique is not evolving any more, and that the complications, both early and late, are well known, as well as their chances of occurrence [7].  We may speculate that the situation is similar concerning TURP – the technique is old enough to be a gold standard, and it does not evolve any more.

In a study published in 1998, Mearini stresses that open surgery still has a strong indication in the treatment of BPH, therefore every urologist needs to develop its skills in traditional surgery [8]. But, because the incidence of large prostates is decreasing, so is the experience of the new generation of urologists, more used with endoscopy and more prone to using it more and more.

In 2001 Tubaro published his observations on a relatively small series of open prostatectomies, for prostates averaging 63g at enucleation, concluding that open prostatectomy offers probably the maximum obtainable relief of obstruction and therefore should be considered the reference standard for evaluating all other treatments for BPH [9]. 

In a study published in 1999 in California, the authors observe that TURP has significantly developed in the 1990s, offering significantly lower perioperative and postoperative complication rates than before, while improving efficacy. At the same time, the average hospital stay and catheterization time have constantly decreased in time [10].

Although the decision between open or endoscopic surgery for BPH seems clear based on the recommendations from the Guidelines, there are several aspects that may change things in daily practice. In the first aspect there are the personal options of the urologist and the patient for one or the other technique. Strong arguments in favor of TURP are the good results in the long run, along with shorter hospital stay and catheterization times.

## Conclusions

Although newer and more promising techniques are readily available in the daily practice, TURP and open surgery are still the only reliable methods for performing a correct and complete prostatectomy in patients with large prostates.

Data in the literature clearly states that, for prostates over 80ml, the open technique should be the treatment of choice, due to the increased rate of complications and better overall outcome. Despite the numerous studies supporting the recommendations, there is enough data to support TURP for large prostates, taking into account some special indications or other particular aspects that lead to this modified attitude.

Nevertheless, our study proves that, in experienced hands, using the proper technique, TURP may lead to similar results, while keeping the complication rate low and obtaining shorter hospitalization times and lower costs. It is clear for us that TURP for large prostates is an intervention not to be performed by the beginner endoscopist, nor by the faint hearted. But, with adequate training and step by step approach, it becomes a safe, clean and efficient technique.

Our study compares TURP with open prostatectomy, for large sized prostates. Nevertheless, the results sustain the idea that, in experienced hands, TURP is equally effective for both mid–sized and large prostates.

The main disadvantages we encountered after TURP were a slightly higher rate of urinary incontinence and urethral strictures. We consider that these are known outcomes after TURP, so that our results can be interpreted as normal, and not particular due to a larger prostate. All our results indicate that, even for large prostates, TURP may lead to the same results it would have for a prostate smaller than 80 ml.

Taking into account all that, we may consider that other non–medical aspects may lead to the decision of operating endoscopically a large prostate, and this includes the necessity for short hospitalization, the increased addressability of the service or several urologists working there, or the continuously increasing patients' option for minimally invasive surgery.

It is also obvious for us that, in the end, the final option is a matter of personal training and experience – there are many urologists who will perform TURP for a large prostate, and there are urologists who will feel safer performing open surgery for a mid-sized prostate. In this aspect, our study proves that the endoscopist who will decide to operate a large prostate is not wrong in his decision.

**Figure 1:**